# Chloramyl, a New Anæsthetic and an Improved Inhaler

**Published:** 1878-12

**Authors:** George E. Sanford


### ARTICLE V.

### *Chloramyl, a new Anæsthetic and an Improved Inhaler.*

BY GEORGE E. SANFORD, M. D.

Having had considerable experience in the administration of the various anæsthetics in use at the present day, viz: chloroform, ether, etc., and not feeling satisfied with the safety, or rather *unsafety* of chloroform, or with the many faults of sulphuric ether, which so nearly counterbalance its comparative safety as to preclude its use in favor of chloroform in many cases, I have therefore experimented with various compounds in the hope of discovering a new and better anæsthetic.

Early in the month of April, 1877, while treating an asthmatic patient with the nitrite of amyl, I became impressed with the idea of augmenting the heart's action with the drug, and thereby *preventing* the tendency to syncope and asphyxia, from paralysis of the heart, in cases of chloroform narcosis.

I then began a series of experiments upon animals, first administering chloroform and then the nitrite of amyl. Then I began to mix them for use, aiming to get such a proportion of the amyl as would just counteract the paralytic effect of the chloroform. I found that while pure chloroform (Squibbs') would mix readily with the nitrite of amyl, producing a fine clear solution, the chloroform of other manufacturers was unsatisfactory, leaving a milky solution of unpleasant odor. Continuing my experiments, I came to the conclusion that a quarter of an ounce of the nitrite of amyl to the pound of Squibbs' chloroform was about the proper strength, and that the combination was far safer for general anæsthetic purposes than chloroform uncombined; indeed, in my hands, and in those of others, so far as tried, it seems to be fully as safe as sulphuric ether, and far more pleasant in its administration, possessing all of the advantages of pure chloroform, but without its dangers.

Upon becoming satisfied of the value of my discovery, I named it chloramyl. I first administered chloramyl to persons in June, 1877, as follows: June 6th I administered the compound to Charles Detric, a young, healthy man, for the purpose of dressing a badly crushed thumb, both the patient and bystanders being wonderfully pleased with its operation. Next, June 16th, I gave it for amputation of a finger; then, June 18th, for extraction of a tooth; since which time I have employed it (chloramyl) in a great variety of cases in both surgical and obstetrical practice. I find that patients usually take it better than chloroform alone, and so far there has not been the first indication of danger from its use. In exhibiting chloramyl, the patient's face becomes flushed much sooner than with chloroform; but press the drug right along and the countenance does not become pale. Both the heart's action and respiration are kept up thoroughly throughout the anæsthesis. I have given this prescription to several physicians, and induced them to try the chloramyl with the most satisfactory results. I have also (last month) communicated the same to Professors Maclean, Dunster, and Frothingham, of Michigan University; and have reported my discovery to the Cayuga County, N. Y. Medical Society.

Having noticed lately several communications in the colums of the *Record*, from Dr. F. A. Burrall and others, on the use of amyl nitrite as an antidote to chloroform in cases of poisoning, I concluded to publish my discovery. As Dr. Burrall states in his article in the *Record* of July 20th, "With our present knowledge of the antidotal properties of amyl nitrite in relation to chloroform, it is but justice to our patients to have it at hand when chloroform is administered." I agree with him that we should have it at hand, but not in a separate bottle, to use *after the danger has become iminent*, but (as it produces no ill effects) *mixed with the chloroform*, in such a proportion as to prevent the approach of danger, by both syncope and asphyxia; for such I claim to be the effect of this combination, and as such as I give chloramyl to the medical profession, asking that it may be given a full and fair trial, and trusting that it may become the humble instrument in other hands of saving human life. Not that I would detract from the honors due the inventor of chloroform, for it was a grand invention; but if we can relieve its administration from the embarrassment and danger which have heretofore attended its use, will it not indeed be a great boon to humanity?

The formula I use for chlorymal is

℞. Squibb's chloroform - - - 1 lb.
Nitrite of amyl - - - - 2 drachms.
Mix.

But I would suggest that the amount of nitrite of amyl be diminished in long and tedious operations, and on further trial it may prove best to vary the proportions, the point aimed at being to use just sufficient amyl nitrite, to counteract the paralytic effect of the chloroform.—*Medical Record.*

---

